# Dual ligand-targeted Pluronic P123 polymeric micelles enhance the therapeutic effect of breast cancer with bone metastases

**DOI:** 10.32604/or.2023.044276

**Published:** 2024-03-20

**Authors:** HUAN GAO, JIE ZHANG, TONY G. KLEIJN, ZHAOYONG WU, BING LIU, YUJIN MA, BAOYUE DING, DONGFENG YIN

**Affiliations:** 1 Jiaxing Key Laboratory for Photonanomedicine and Experimental Therapeutics, Department of Pharmaceutics, College of Medicine, Jiaxing University, Jiaxing, 314001, China; 2 Department of Pharmacy, The General Hospital of Xinjiang Military Region, Urumqi, 830000, China; 3 Department of Pathology and Medical Biology, University Medical Center Groningen, University of Groningen, Groningen, 9713 GZ, The Netherlands; 4 Department of Pathology, Laboratory of Experimental Oncology, Erasmus MC, Rotterdam, 3015 GD, The Netherlands; 5 Department of Pharmacy, Jiaxing Maternal and Child Health Care Hospital, Affiliated Hospital of Jiaxing University, Jiaxing, 314001, China; 6 Qinghai Enlu Biotechnology Co., Ltd., Haidong, 810700, China

**Keywords:** Pluronic micelles, Targeted nanotherapeutics, Nanoparticulate drug delivery system, Breast cancer with bone metastasis, Therapeutic efficacy

## Abstract

Bone metastasis secondary to breast cancer negatively impacts patient quality of life and survival. The treatment of bone metastases is challenging since many anticancer drugs are not effectively delivered to the bone to exert a therapeutic effect. To improve the treatment efficacy, we developed Pluronic P123 (P123)-based polymeric micelles dually decorated with alendronate (ALN) and cancer-specific phage protein DMPGTVLP (DP-8) for targeted drug delivery to breast cancer bone metastases. Doxorubicin (DOX) was selected as the anticancer drug and was encapsulated into the hydrophobic core of the micelles with a high drug loading capacity (3.44%). The DOX-loaded polymeric micelles were spherical, 123 nm in diameter on average, and exhibited a narrow size distribution. The *in vitro* experiments demonstrated that a pH decrease from 7.4 to 5.0 markedly accelerated DOX release. The micelles were well internalized by cultured breast cancer cells and the cell death rate of micelle-treated breast cancer cells was increased compared to that of free DOX-treated cells. Rapid binding of the micelles to hydroxyapatite (HA) microparticles indicated their high affinity for bone. P123-ALN/DP-8@DOX inhibited tumor growth and reduced bone resorption in a 3D cancer bone metastasis model. *In vivo* experiments using a breast cancer bone metastasis nude model demonstrated increased accumulation of the micelles in the tumor region and considerable antitumor activity with no organ-specific histological damage and minimal systemic toxicity. In conclusion, our study provided strong evidence that these pH-sensitive dual ligand-targeted polymeric micelles may be a successful treatment strategy for breast cancer bone metastasis.

## Introduction

Breast cancer is the most common malignant tumor among women worldwide. There were 2.3 million women diagnosed with breast cancer and 685,000 deaths globally in 2020 [1]. Patients with breast cancer frequently suffer from metastatic relapse months to decades after the initial diagnosis and treatment, which dramatically decreases their life expectancy. The organ in which breast cancer metastasis most commonly occurs is bone [2], with bone metastasis secondary to breast cancer occurring in approximately 70% of patients with advanced disease [3]. Bone metastases are generally osteolytic, as cancer cells obstruct the normal bone remodeling process and enhance osteoclast-mediated bone resorption [4]. As a result, patients with bone metastases often suffer from pain and are at risk for skeletal complications such as fractures, hypercalcemia, and spinal cord compression, which all substantially decrease their quality of life [5,6].

Despite advances in cancer treatment, therapeutic options for bone metastases remain inadequate and generally palliative [7,8]. Current treatments aim to inhibit tumor growth and bone resorption. Inhibiting tumor growth remains a challenge, as many anticancer agents, such as chemotherapy drugs and radiopharmaceuticals, do not reach the bone in efficacious concentrations due to the hardness, poor permeability, and physiological and biochemical processes of bone [9]. Increasing the dose of these drugs to achieve therapeutic effects is generally not possible because it leads to grievous systemic side effects due to their lack of tissue specificity [10,11]. Slowing bone resorption, in contrast, is easier to establish by inhibiting the activity of osteoclasts, although this process has smaller effects on tumor growth. Therefore, a bone-targeted drug delivery system to simultaneously inhibit tumor growth and bone resorption would be a major advance in the treatment of bone metastases.

Polymeric micelles as drug carriers have been demonstrated to be promising in cancer chemotherapy and are already under clinical evaluation [12–14]. In our previous work [15], we synthesized polymeric micelles using Pluronic P123 (P123; polyoxyethylene-polyoxypropylene-polyoxyethylene triblock copolymer). We chose P123 due to its commercial availability, biocompatibility, and safety profile [16]. The micelles can be easily prepared through the self-assembly of amphiphilic copolymers, which results in the formation of a core-shell structure. The hydrophilic shell makes the micelles water soluble, allowing for intravenous delivery, while the hydrophobic core carries the drug payload for therapy. The micelles provide various advantages, including drug solubilization, controlled drug release, escape from reticuloendothelial system uptake, and passive tumor targeting by enhanced permeability and retention effects [17,18]. In addition, Pluronic molecules can inhibit the release of the drug-removal protein P-glycoprotein, which hinders the distribution of many drugs to multidrug-resistant tumors [19]. Moreover, active targeting can be achieved by conjugating target-specific moieties (ligands) to the terminal hydroxyl groups of P123 onto the surface of micelles.

Bisphosphonates are a group of drugs that prevent the loss of bone density by inducing apoptosis in osteoclasts and can be used as bone-targeting ligands. It has been shown that drugs or drug carriers conjugated with alendronate (ALN), a second-generation bisphosphonate, accumulate to a greater extent in bone than in healthy tissues [20,21]. Additionally, the accumulation of ALN is 10- to 20-fold higher in the bone tumor environment than in healthy bone due to the extensive bone remodeling by cancer cells [22–24]. Therefore, in this work, to improve the selectivity of P123 micelles to breast cancer bone metastases and simultaneously inhibit bone resorption, ALN was used to modify P123 micelles. To further target breast cancer cells while also decreasing the toxicity to normal cells, DMPGTVLP fused to the N-terminus of the p8 phage protein (DP-8) was added as a second ligand. This cancer-specific phage protein was isolated from the 8-mer (f8/8) phage landscape library by screening against MCF-7 breast cancer cells [25]. DP-8 is able to bind to nucleolin [25,26], a multifunctional protein involved in several cellular processes that has been shown to be overexpressed on the surface of breast cancer cells and different other types of cancer cells [27,28]. One of the diverse roles of nucleolin is to serve as a shuttling protein between the cytoplasm and nucleus, which is one of the mechanisms underlying the extracellular regulation of nuclear events [26]. It was shown that DP-8 enhanced the accumulation and antitumor activity of nanomedicines in mouse models of breast cancer [29,30].

In this study, to enhance the therapeutic effect of doxorubicin (DOX) on breast cancer bone metastases, P123 micelles conjugated with ALN and DP-8 (P123-ALN/DP-8) were prepared by the thin-film hydration method, in which ALN, DP-8, and Pluronic were linked by amide bonds. ALN and DP-8 were innovatively selected as organic drug ligands to deliver chemotherapeutic drugs targeting DOX to bone metastasis sites. The antitumour effects and antibone resorption effects of bone-targeting nanoparticles were further investigated *in vitro* and *in vivo*.

## Materials and Methods

The materials used in this study were DOX hydrochloride (purity 99%) (Wuhan Dahua Weiye Pharmaceutical Chemical, Wuhan, China); P123 (Sigma‒Aldrich, St. Louis, MO, USA); ALN, DP-8, N-hydroxysulfosuccinimide sodium salt (NHS), bovine serum albumin (BSA), 3-(4,5-dimethylthiazol-2-yl)-2,5-diphenyl tetrazolium bromide (MTT), 4′,6-diamidino-2-phenylindole (DAPI), and Neutral Red (NR) dye (Shanghai Titan Technology Company, Shanghai, China); Dulbecco’s modified Eagle medium (DMEM), fetal bovine serum (FBS), phosphate buffered solution (PBS), 0.25% trypsin, and penicillin‒streptomycin solution (Gibco, Thermo Fisher Scientific, Waltham, MA, USA); Annexin V-FITC Apoptosis Detection Kit and PI/RNase Staining Buffer Reagent test kit (BD Biosciences, Franklin Lakes, NJ, USA); HA microparticles (Shanghai Aladdin Biochemical Technology, Shanghai, China); and Western blot reagents (Beyotime, Shanghai, China). All other chemicals and reagents used were of analytical grade or higher and commercially obtained.

### Cell culture

Human triple-negative breast cancer (MDA-MB-231) cells were purchased from Nanjing KGI Biotechnology Development Company (Jiangsu, China).

### Animals

Female athymic BALB/c-nu/nude mice (aged 4–6 weeks, 18 ± 2 g) and female Sprague-Dawley rats (aged 5–7 days, 2 ± 1 g) were purchased from the Suzhou Cavins Park Model Animal Research Company (Jiangsu, China). All animal procedures were performed in accordance with protocols approved by the Animal Care and Use Committee, Jiaxing University (approval number: JUMC2022-152).

### Synthesis of P123-ALN/DP-8

First, the Pluronic P123 copolymer was activated using NHS. Briefly, P123 copolymer (3.468 g, 0.6 mmol) and triphosgene (0.3560 g, 1.2 mmol) were dissolved in 30 mL of a solution of anhydrous toluene and anhydrous dichloromethane (2:1) at 25°C and stirred at 500 rpm overnight. After evaporating, the residue was dissolved in a 20 mL solution of anhydrous toluene and anhydrous dichloromethane (2:1). Next, NHS (0.1370 g, 1.2 mmol) and anhydrous triethylamine (0.2 mL) diluted with anhydrous dichloromethane (1 mL) were added dropwise and stirred at 200 rpm for 4 h. After the reaction was completed, the solution was filtered. The residue was dissolved in 100 mL ethyl acetate at 50°C and then filtered again. Ethyl acetate was removed from the solution by rotary evaporation. The solidified reaction product (named P123-NHS) was cooled and stored in dry conditions at −20°C.

Second, ALN and DP-8 were conjugated to the P123 copolymer. Briefly, ALN (0.3900 g, 1.2 mmol) or DP-8 (0.85 g, 1.2 mmol) was slowly added to P123-NHS (0.85 g, 1.2 mmol) dissolved in 10 mL of PBS. The mixture was stirred at 200 rpm for 24 h under a nitrogen atmosphere. Then, the product was dialyzed in deionized water for 72 h, where the aqueous phase was changed every 24 h. Last, the product was lyophilized and stored at −20°C.

### Preparation of micelles

The thin film hydration method was applied to prepare the P123-ALN/DP-8@DOX [15]. In brief, 5.48 mg of DOX•HCl was added to 7.28 mL of methanol solution, then three times the molar amount of triethylamine was added, and the mixture was stirred magnetically overnight at room temperature to achieve full dissolution. Next, 50 mg of P123, 40 mg of P123-ALN and 10 mg of P123-DP-8 polymer material were added, and the solvent was removed by rotary evaporation. After that, 8.58 mL of sterilized water for injection was added for hydration and mixed with magnetic stirring for 30 min. The solution was further filtered with a 0.22-μm microporous membrane filter and freeze-dried overnight to prepare P123-ALN/P123-DP-8@DOX, which was stored at −20°C for further utlize. P123@DOX micelles were prepared and stored in the same manner.

### Characterization of P123-ALN/DP-8@DOX micelles

The morphology of the micelles was observed by transmission electron microscope (JEOL, Tokyo, Japan). The micelles were dissolved in an appropriate amount of distilled water, and a drop of solution was placed on a copper mesh for observation, after which phosphotungstic acid was reduced. A nanoparticle size potentiometer (Mastersizer 3000, Malvern Instruments, Worcestershire, UK) was used to measure the particle size and zeta potential.

### Determination of drug entrapment efficiency (EE) and DOX loading (DL) capacity

The concentration of entrapped DOX was determined by high-performance liquid chromatography (HPLC) using a Waters 2487 system (Waters Corporation, Milford, MA, USA) equipped with a C_18_ column (4.6 mm × 250 mm), and the mobile phase consisted of a buffer (1.44 g of sodium dodecyl sulfate and 0.68 mL of phosphoric acid dissolved in 500 mL of water), acetonitrile and methanol (40:54:6, v:v:v), delivered at a flow rate of 1.0 mL/min. The injection volume was 20 μL. The detection wavelength was 254 nm, and the column temperature was 25°C. The concentration of DOX was determined based on the peak area. The encapsulation rate and drug load were calculated according to the following formulas:

EE% = (measured amount of DOX)/(amount of DOX added) × 100%

DL% = (weight of DOX in the micelle)/(weight of the micelle) × 100%

### In vitro DOX release

The release of DOX from the micelles was analyzed by the dialysis method at different pH values (pH = 5.0, 6.8, and 7.4) with 1% Tween-80. Briefly, 2 mL of P123-ALN/DP-8@DOX micelles were separately dispersed in 20 mL of PBS buffer and loaded into a dialysis bag (molecular weight-cutoff of 3.0 kDa). The release system was kept at 37°C under continuous stirring at 100 rpm, and 2 mL of release medium was collected with an equal volume of fresh PBS added at the predetermined time points (1, 2, 4, 8, 12, 24, 48, 72, 96 and 120 h). The cumulative released DOX in PBS was analysed by HPLC. The measurement was conducted in triplicate.

### HA binding assay

The affinity of micelles for bone was evaluated using HA particles. HA microparticles (100 mg) were mixed with 10 mL of free DOX, P123@DOX, or P123-ALN/DP-8@DOX (final DOX concentration of 400 μg/mL) dissolved in PBS (pH = 7.4) and vortexed. HA microparticles were allowed to settle, and 2 mL supernatant was withdrawn for analysis at predetermined time intervals (15, 30, 60, and 90 min). The absorbance of suspensions at 589 nm before (A_before_) and after (A_after_) mixing with HA microparticles was recorded using a fluorescence spectrophotometer (Hitachi, Tokyo, Japan). The following equation was used to determine the adsorption rate: Adsorption rate% = (A_before_ − A_after_)/A_before_ × 100%

### In vitro uptake analysis (confocal microscopy and flow cytometry)

The internalization and intracellular localization of P123-ALN/DP-8@DOX were evaluated by confocal microscopy. MDA-MB-231 cells (cell concentration of 1 × 10^4^/mL, volume of 500 μL) were seeded in a small confocal dish and placed at 37°C in a 5% CO_2_ incubator for 24 h. Then, free DOX, P123@DOX, and P123-ALN/DP-8@DOX solutions were added at a final DOX concentration of 10 µg/mL in each well. After 0.5 and 2 h of incubation, the cells were fixed with 0.5 mL of 4% paraformaldehyde and stained with 200 µL of DAPI (100 ng/mL in Milli-Q water) for 15 min. Finally, the cells were observed by confocal microscopy imaging using the Leica TCS SP2 system (Leica, Heidelberg, Germany). DAPI and DOX were excited at 405 and 620 nm, respectively.

Quantification of cellular uptake was assessed using flow cytometry to confirm the internalization of the treatments.MDA-MB-231 cells (cell concentration of 1 × 10^4^/mL, volume of 2 mL) were inoculated in a six-well plate and placed at 37°C in a 5% CO_2_ incubator for 24 h. Then, free DOX, P123@DOX, and P123-ALN/DP-8@DOX solutions were added at a final DOX concentration of 10 µg/mL in each well. After 0.5 and 2 h of incubation, the cells were washed with PBS, digested with 0.25% trypsin and centrifuged at 800 rpm for 5 min. The supernatant was discarded, and 500 μL of PBS-suspended cells was added for flow cytometry (Sony Biotechnology, Tokyo, Japan). Data were processed and plotted using GraphPad Prism (GraphPad Software, La Jolla, CA, USA).

### In vitro cytotoxicity

The MTT assay is widely used for the study of cell proliferation and cytotoxicity. Briefly, MDA-MB-231 cells (cell concentration of 1 × 10^4^/mL, volume of 100 μL) were seeded in 96-well plates and placed at 37°C in a 5% CO_2_ incubator for 24 h, After washing once with PBS, free DOX, P123@DOX, and P123-ALN/DP-8@DOX solutions were added at a finalDOX concentrations ranging from 0–40 µg/mL in each well and incubated for 48 h under the same conditions. Then, 20 μL of MTT solution (5 mg/mL) was added to each well and incubated for another 4 h., Afterwards, the medium was replaced with 300 μL of DMSO. The optical density (OD) of each well was measured at 490 nm using a BioTek Synergy microplate reader (Bio-Tek Winooski, Vermont, USA). The cell viability was determined with the following equation:

cell viability (%) = (OD _sample_ – OD_blank_)/(OD _control_ – OD_blank_) × 100%

The 50% inhibitory concentration (IC_50_) was calculated from the cell viability at the corresponding concentration using GraphPad Prism.

### Analysis of the cell apoptosis mode and Western blotting

The Annexin V-FITC/PI Apoptosis Detection Kit is a universal reagent kit for detecting apoptosis. Briefly, MDA-MB-231 cells (cell concentration of 1 × 10^4^/mL, volume of 2 mL) were seeded in a six-well plate and placed at 37°C in a 5% CO_2_ incubator for 24 h. Then, free DOX, P123@DOX, and P123-ALN/DP-8@DOX solutions were added at a final DOX concentration of 10 µg/mL in each well. After culturing for 48 h, the cells were trypsinized, collected in 1.5-mL sterile centrifuge tubes and incubated with 5 µL annexin V-FITC (100 ng/mL) in the dark for 10 min. Then, 5 µL of propidium iodide (PI) was added to each group before analyzed by flow cytometry (Sony Biotechnology, Tokyo, Japan). Untreated cells were used as a control. The tests were conducted in triplicate, and the apoptosis rate was determined by FlowJo software (BD Biosciences, San Jose, CA,USA).

For Western blotting, MDA-MB-231 cells were seeded and treated as described in the “Apoptosis assay” section. The cells were collected and lysed for 30 min on ice with RIPA buffer containing a cocktail of protease and phosphatase inhibitors. After centrifugation at 15,000 rpm for 20 min at 4°C, the supernatant was collected. The protein concentrations of the supernatant were measured using a Nanodrop spectrophotometer (Thermo Fisher Scientific, Waltham, MA, USA). The proteins were obtained by SDS‒PAGE (5% acrylamide, 10% SDS, 6 μg/μL protein concentration, 10 µL/well loading amount, running voltage 80 V for 30 min, then 120 V for 90 min). Electrotransferred to polyvinylidene fluoride (PVDF) membranes (Thermo Fisher Scientific, 220 mA, 120 min, 4°C) and blocked with QuickBloc Blocking Buffer (Beyotime, Shanghai, China) for 10 min at RT. The blocked PVDF membranes containing the proteins were incubated with primary antibodies against caspase-3 (Abcam, Cambridge, MA, USA) and β-actin (Abcam, Cambridge, MA, USA) overnight at 4°C. Then, the membranes were washed and incubated with horseradish peroxidase-linked secondary antibodies (Abcam, Cambridge, MA, USA) for 1 h at RT. The target protein bands were visualized by enhanced chemiluminescence (Bio-Rad ChemiDoc XRS imaging system, Hercules, CA, USA). β-actin was used as a loading control.

### In vitro bone binding analysis

The parietal bones from rats were used to further illustrate the specific targeting of bone tissue by P123-ALN/DP-8@DOX micelles. Five- to seven-day-old Sprague-Dawley rats were sacrificed, and their parietal bones, including the surrounding excess tissue, were dissected under sterile conditions. The bones were washed with normal saline until they were clean. Then, the bones were washed twice with PBS buffer. One piece of bone was placed into each of the wells of a six-well plate with DMEM containing 10% FBS and 5 mg/mL BSA. The medium was replaced with fresh medium containing free DOX, P123@DOX, or P123-ALN/DP-8@DOX at a final DOX concentration of 10 µg/mL for 48 h. Next, the bones were removed and split in two. Half of the bones were fixed with 2.5% glutaraldehyde for 2 h, dehydrated in a series of tert-butyl alcohol solutions (50%, 70%, 95%, and 100%) for 15 min, and gold-coated. Field emission scanning electron microscopy (JEOL, Tokyo, Japan) was used to observe free DOX, P123@DOX, and P123-ALN/DP-8@DOX in the bone tissue.

### Antitumor, antiosteoclastogenic, and anti-bone resorption activities in an in vitro 3D bone metastasis model of breast cancer

To further evaluate the effects of P123-ALN/DP-8@DOX on bone metastases, an *in vitro* 3D model was constructed using the parietal bones of 5- to 7-day-old Sprague-Dawley rats to simulate the *in vivo* bone metastasis microenvironment. In brief, parietal bones were obtained as described in the section on bone targeting. One piece of bone was placed into a six-well plate and incubated with complete DMEM for 24 h. Then, MDA-MB-231 cells were seeded at a density of 1 × 10^5^ cells/well and coincubated with the bones under standard culture conditions. At predetermined intervals of 0, 2, 4, and 6 days, the bones were removed, fixed with 2.5% glutaraldehyde for 2 h, dehydrated in a series of tert-butyl alcohol solutions of 50%, 70%, 95%, and 100% for 15 min, and gold-plated. Field emission scanning electron microscopy (JEOL, Tokyo, Japan) was used to observe the bone tissue tumor cells.

After the successful establishment of a three-dimensional *in vitro* model of bone metastasis from the breast cancer, parietal bone was obtained, and MDA-MB-231 cells were incubated for 6 days in standard culture conditions. Then, the medium was replaced with fresh medium containing free DOX, P123@DOX, or P123-ALN/DP-8@DOX at a final DOX concentration of 10 µg/mL and incubated for 48 h. The bones were removed and split in two, and the following experiments were performed: (1) Half of the bones were fixed with 2.5% glutaraldehyde for 2 h and dehydrated in a series of tert-butyl alcohol solutions of 50%, 70%, 95%, and 100% for 15 min. The dehydrated bones were dried on a freeze-drying device and then gold-plated. Afterward, the tumor cells in the bone tissue were observed by SEM. (2) The other bones were incubated with DMEM supplemented with 70 μg/mL NR for 1 h under standard culture conditions. In this live bone culture approach, NR was rapidly taken up by osteoclasts [31]. The number of osteoclasts was quantified using an optical CH-2 microscope (Olympus, Tokyo, Japan). Osteoclasts could be well distinguished from other osteocytes and stroma because they take up NR and are large and multinucleated. After NR staining and microscopic examination, the bone resorption area within the same bone was quantified. The bones were washed in PBS, fixed in 2.5% glutaraldehyde for 12 h, and counterstained with 1% silver nitrate solution for 30 min in the dark. The extent of bone resorption was assessed under the microscope where resorption regions were transparent to light.

### In vivo biodistribution analysis

The breast cancer bone metastasis model was established by direct injection of MDA-MB-231 cells into the bone marrow cavity in the left tibia of mice [32–34]. Briefly, BALB/c-nu/nu mice were anesthetized with 10% (w/v) chloral hydrate. Then, MDA-MB-231 cells (1 × 10^6^ cells in 100 μL of PBS) were injected into the left tibia with a 23-gauge needle. On the fourteenth day after the MDA-MB-231 cells were implanted, the mice received 200 μL of saline, free DOX, P123@DOX or P123-ALN/DP-8@DOX micelles at a final DOX concentration of 5 mg/kg body weight by tail vein injection. At predetermined time intervals of 2, 6, 12, and 24 h, the distribution of DOX in the tumor-bearing mice was observed by using a whole body imager (Shanghai Instrument Experiment Factory, Shanghai, China; λ_ex_ = 480 nm; λ_em_ = 620 nm).

### In vivo antitumor efficacy and safety

About 24 tumor-bearing mice were randomly divided into four groups: saline, free DOX, P123@DOX, and P123-ALN/DP-8@DOX. Then, the mice were injected intravenously with saline, free DOX, P123@DOX, P123-ALN/DP-8@DOX at a final concentration of 5 mg/kg DOX, g and 200 µL injection volume or saline via the tail vein once a week for 2 weeks. The general health of the mice was monitored daily during the experiment. The animals were weighed, and tumor length (L) and width (W) were measured with a Vernier caliper every 2 days. The tumor volume (V) was calculated by V = 0.5 [L × W^2^]. At the end of the experiment, plasma samples were collected from the eyeball vein for the determination of white blood cell (WBC) numbers, alanine aminotransferase (ALT) and creatinine (Cr) levels. The mice were sacrificed by cervical dislocation, and the liver, spleen, heart, kidneys, lungs, and left hind leg were dissected. Fluorescence of the excised organs was imaged using an IVIS Spectrum small animal imaging system (PerkinEImer, Waltham, MA, USA; λ_ex_ = 480 nm; λ_em_ = 620 nm). The tissues were fixed with 10% formalin (Beyotime, Shanghai, China) for 48 h, embedded in paraffin, and cut into 5-μm sections. The sections were stained with a hematoxylin and eosin (H&E) staining kit (Abcam, Cambridge, MA, USA) and imaged under a light microscope (Leica, Wetzlar, Germany). Tumor tissue of the left hind limb was separated and weighed. The tumor growth inhibition rate (IRT) was calculated according to the following formula:

IRT% = (tumor weight_control_ – tumor weight_treated_)/tumor weight_control_ × 100%

### Statistical analysis

Statistical analysis was performed in GraphPad Prism (GraphPad Software, San Diego, CA, USA) and SPSS (Statistical Package for Social Sciences; IBM, Chicago, IL, USA). Values are given as the mean ± standard deviation (SD). Student’s *t* test was used for intergroup analysis. A *p* value of 0.05 was considered statistically significant.

## Results and Discussion

### The Characterization of P123-ALN/DP-8@DOX micelles

**Figure 1:**
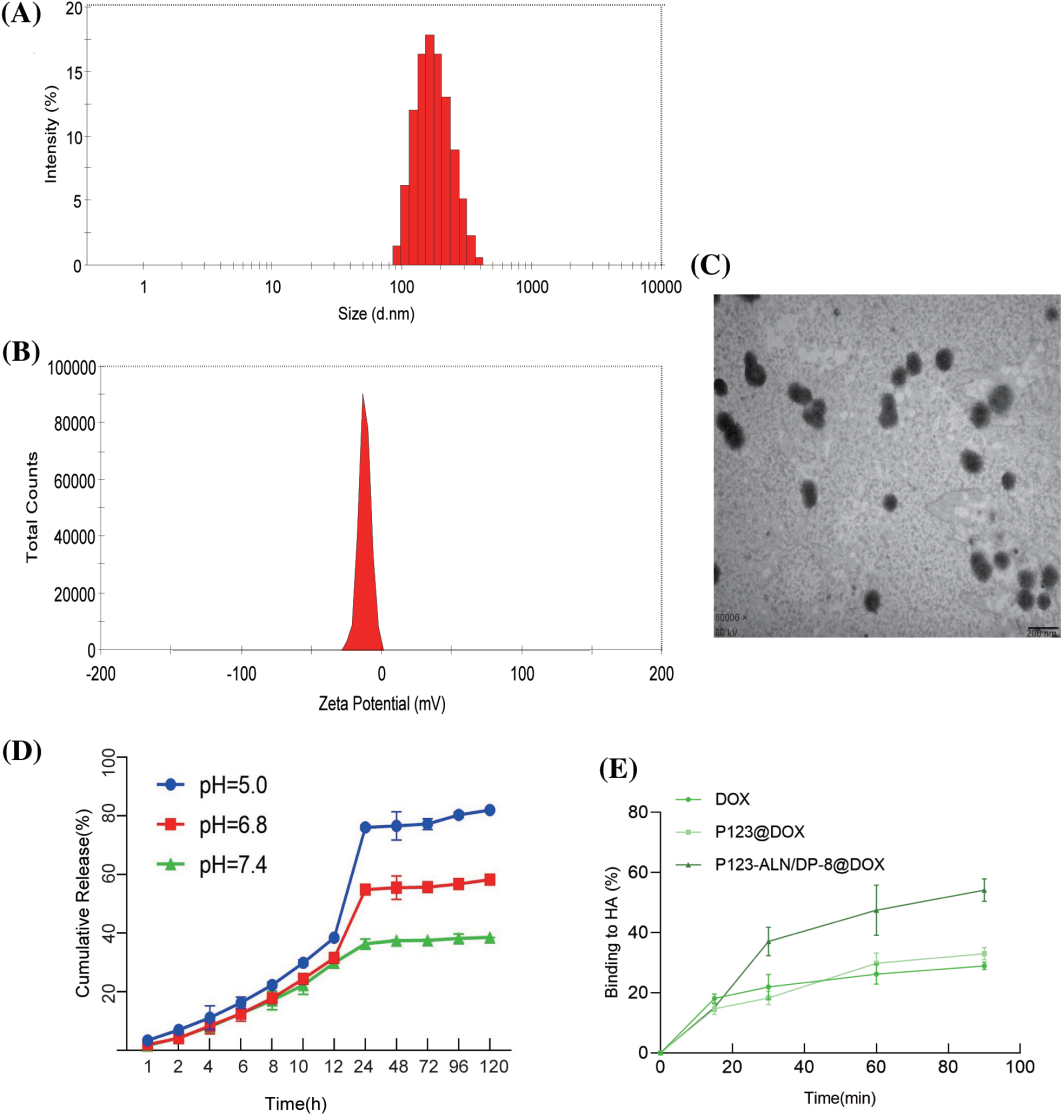
Characterization of P123-ALN/DP-8@DOX micelles. (A) Size distribution and (B) Zeta potential measured by dynamic light scattering and electrophoretic mobility analysis, respectively. (C) Transmission electron microscopy image of P123-ALN/DP-8@DOX micelles. The scale bar is 200 nm. (D) *In vitro* DOX release from P123-ALN/DP-8@DOX micelles at 37°C under different pH conditions. (E) The adsorption of free DOX, P123@DOX, and P123-ALN/DP-8@DOX to hydroxyapatite (HA). Data are expressed as the mean ± SD (n = 3).

The particle size and size distribution of P123-ALN/DP-8@DOX micelles are shown in Fig. 1A. The diameter of the micelles was 122.97 ± 4.72 nm. P123-ALN/DP-8@DOX micelles were in a size range suitable for accumulation in tumor tissue through the enhanced permeability and retention effect [35]. The zeta potential of P123-ALN/DP-8@DOX micelles was −12.60 ± 1.90 mV, as shown in Fig. 1B. The negative surface charge of the micelles was attributed to the presence of ALN and DP-8 [36]. A TEM image of P123-ALN/DP-8@DOX is depicted in Fig. 1C, displaying micelles with a spherical morphology and a smooth surface. The micelles showed a high EE and DL capacity of 76.87% ± 9.72% and 3.44% ± 0.69%, respectively, and DOX was tightly wrapped in the hydrophobic core.

### In vitro DOX release

The release of DOX from P123-ALN/DP-8@DOX was investigated in PBS containing 1% Tween-80 under simulated normophysiological conditions (pH = 7.4) and an acidic microenvironment (pH = 6.8 and 5.0) at 37°C. As shown in Fig. 1D, the rate and amount of DOX released from the micelles were pH-dependent, with faster DOX release at pH 5.0 and 6.8 than at pH 7.4. In an acidic environment, the cleavage of hydrogen bonds within micelles in response to an acidic environment enables the rapid release of DOX. These chemical environment-dependent release kinetics are favorable for a drug delivery system [37], as it is expected that the micelles will be stable in the blood circulation (pH 7.4). Once the micelles are internalized in the endolysosome of cancer cells, DOX will be released from the micelles due to the acidic microenvironment of the endolysosome and diffuse through the cytoplasm to the nucleus [38].

### Hydroxyapatite affinity assay

High affinity to bone is essential for targeted drug delivery to metastatic bone tissue. There is ample evidence that ALN has a strong affinity for bone and is able to attach to hydroxyapatite binding sites on bone surfaces, especially bone surfaces that undergo remodeling due to the increased osteoclast activity in metastatic bone tissue [39–42]. To evaluate the binding capacity of P123-ALN/DP-8@DOX to bone, an HA affinity assay was carried out *in vitro*. As demonstrated in Fig. 1E, after 30 and 90 min of incubation, approximately 38% and 52% of P123-ALN/DP-8@DOX, respectively, was bound with HA. In contrast, after 90 min, 28% and 32% of free DOX and P123@DOX, respectively, were bound to HA. These results indicate that P123-ALN/DP-8@DOX is able to quickly target lytic bone metastases.

### In vitro uptake and intracellular localization

**Figure 2:**
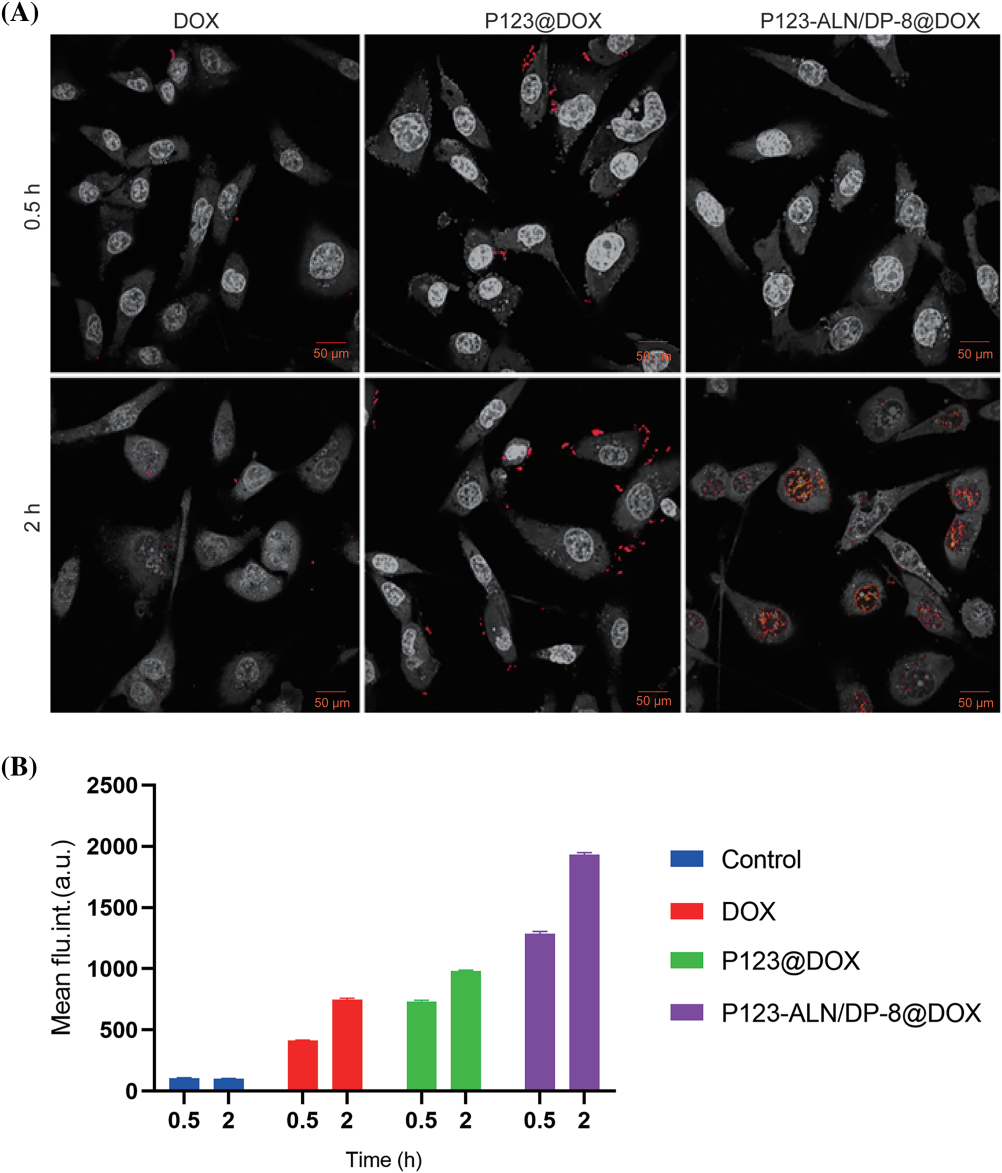
Uptake and intracellular distribution of DOX and DOX-containing micelles in MDA-MB-231 cells. (A) Cells were incubated with free DOX, P123@DOX and P123-ALN/DP-8@DOX at a final DOX (red) concentration of 10 µg/mL for 0.5 and 2 h. DAPI was used to stain the cell nucleus. Cells were imaged by confocal microscopy. Images were edited in Adobe Photoshop for hue, lightness, saturation, and contrast in a clustered manner to preserve relative differences in staining pattern while accentuating the red fluorescence (DOX). (B) Association of DOX, P123@DOX, and P123-ALN/DP-8@DOX with MDA-MB-231 cells as quantified by flow cytometry. Data are expressed as the mean ± SD of DOX fluorescence intensity (n = 3).

The efficacy of drug delivery systems is related to their internalization by cells and the subsequent release of the drug payload. The internalization of P123-ALN/DP-8@DOX by MDA-MB-231 cells and their intracellular localization was investigated by confocal microscopy. The intrinsic fluorescence of DOX was used to image DOX in MDA-MB-231 cells after 2 h of incubation with free DOX, P123@DOX, and P123-ALN/DP-8@DOX (Fig. 2A). Cellular uptake occurred in a time-dependent manner as the fluorescence intensity of DOX increased in all treatment groups when the incubation time increased from 0.5 to 2 h. MDA-MB-231 cells incubated with free DOX for 2 h exhibited very weak fluorescent clusters mainly located in the cytoplasm and to a lesser extent in the nucleus. The fluorescence intensity of DOX increased in cells treated with P123@DOX or P123-ALN/DP-8@DOX compared with cells treated with free DOX, with the strongest fluorescence intensity observed in cells treated with P123-ALN/DP-8@DOX. These results indicated that the addition of both P123 and DP-8 increased the cellular uptake of DOX. Our findings are in line with other studies [41–43]. In general, nanoparticles enter the lysosomes of cells through endocytosis [43,44]. After the cells were treated with P123-ALN/DP-8@DOX for 2 h, DOX fluorescence was almost completely localized in the nucleus of the cells. This finding confirmed that DOX was released from intracellular P123-ALN/DP-8@DOX micelles and had translocated to the nucleus.

The cellular uptake of P123-ALN/DP-8@DOX micelles by MDA-MB-231 cells was further evaluated by flow cytometry, as shown in Fig. 2B. Consistent with the findings of confocal microscopy, the uptake of P123@DOX or P123-ALN/P123-DP-8@DOX by MDA-MB-231 cells was significantly higher than that of free DOX. The highest uptake was observed in cells treated with P123-ALN/DP-8@DOX micelles.

### In vitro cytotoxicity

**Figure 3:**
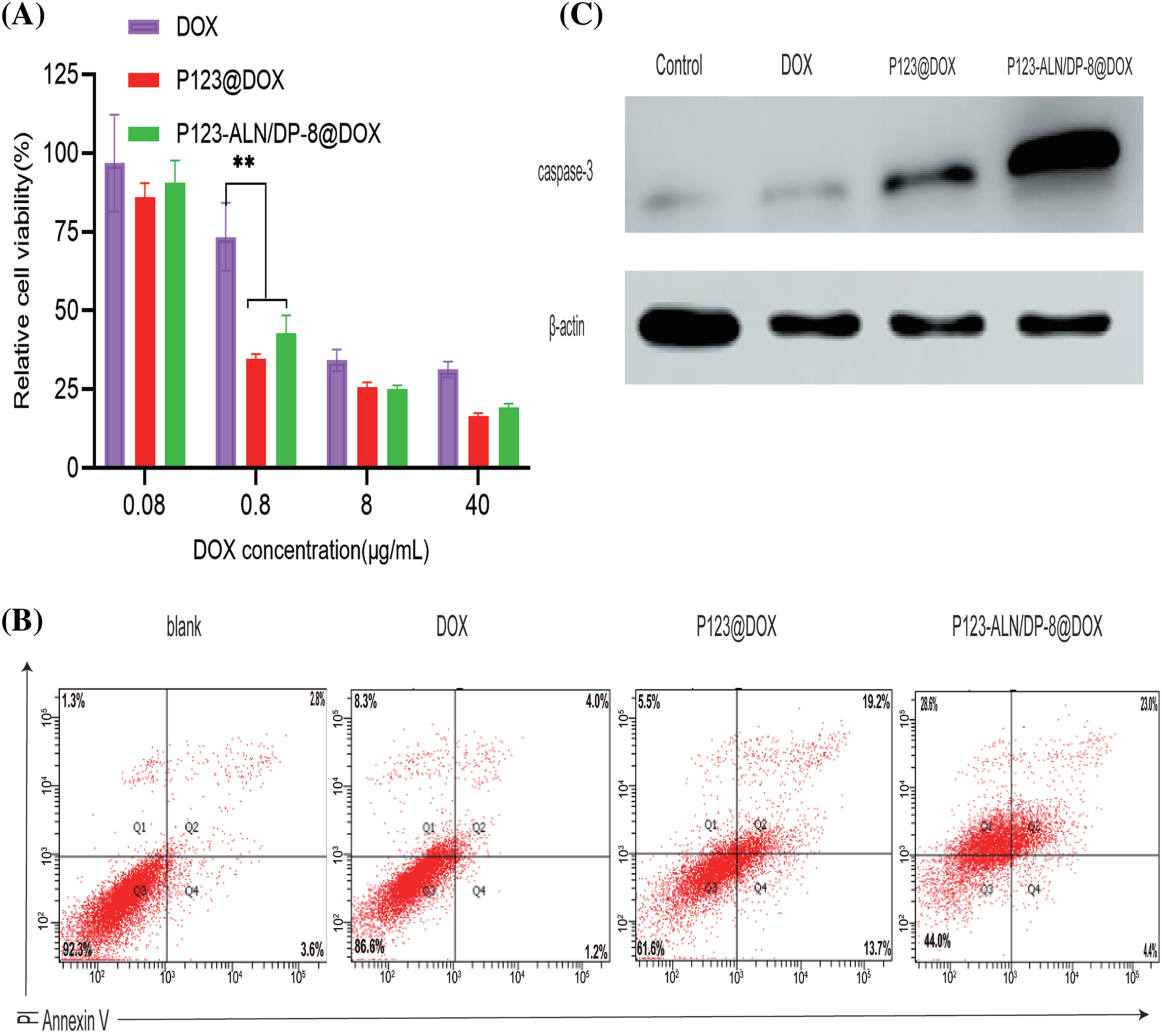
*In vitro* cytotoxicity. (A) Cell viability of MDA-MB-231 cells after incubation with free DOX, P123@DOX, and P123-ALN/DP-8@DOX for 48 h measured with the MTT colorimetric assay. Data were normalized to the average value of the control (untreated) cells. Data are expressed as the mean ± SD (n = 6), **p < 0.01 *vs* control. (B) The mode of cell death was analyzed after staining the cells with FITC-conjugated AV and PI by flow cytometry. The proportion of cells undergoing early apoptosis (AV+/PI−), late stage of apoptosis (AV+/PI+), and necrosis (AV−/PI+) are shown in the lower right quadrant (Q4), upper right quadrant (Q2), and upper left quadrant (Q1), respectively. (C) Protein expression of caspase-3 in MDA-MB-231 cells.

Before transitioning to *in vivo* proof-of-concept studies, the cytotoxicity of P123-ALN/DP-8@DOX towards MDA-MB-231 cells was evaluated via MTT assay. As shown in Fig. 3A, there was no significant difference in the survival rate of cells among the free DOX, P123@DOX and P123-ALN/DP-8@DOX groups when the DOX concentration was 0.08 µg/mL, when the maximum administration concentration was 40 µg/mL, the cell survival rates in the free DOX, P123@DOX and P123-ALN/P123-DP-8@DOX groups were 31%, 17% and 20%, respectively. According to GraphPad Prism 9, the IC50 values of free DOX, P123@DOX and P123-ALN/-DP-8@DOX were 4.69, 0.839, and 0.989 µg/mL, respectively. Both P123@DOX and P123-ALN/DP-8@DOX showed more potent anticancer activity than free DOX. The micelles could enhance the cellular uptake of DOX and increase cytotoxicity, which was verified by laser confocal microscopy and flow cytometry.

### Mode of cell apoptosis

The effect of P123-ALN/P123-DP-8@DOX on MDA-MB-231 cell apoptosis was measured by the Annexin V-FITC/PI double staining method using flow cytometry. The proportions of cells in early (AV+/PI−) and late apoptosis (AV+/PI+) as well as necrosis (AV−/PI+) were determined in the following order: blank cells, cells treated with free DOX, cells treated with P123@DOX, and cells treated with P123-ALN/DP-8@DOX (Fig. 3B). The cell survival rates of cells treated with free DOX, P123@DOX and P123-ALN/DP-8@DOX were 86.6%, 61.6% and 44.0%, respectively. The results were consistent with the cell viability (Fig. 3A) and indicated that the enhanced micellar delivery of DOX potentiated chemotherapeutic efficacy *in vitro*. DOX has been mainly known to mainly induce apoptosis [45]. This experiment confirmed that DOX induced apoptosis independent of the mode of administration, as evidenced by the similar pattern of cell death induced by free DOX and micellar DOX (Fig. 3B). However, there was relatively more necrotic cell death in cells treated with free DOX and P123-ALN/DP-8@DOX.

To verify the mechanism of apoptosis, the changes in the apoptosis-related protein caspase-3 were analyzed by western blotting. The data are summarized in Fig. 3C. Compared with the expression of caspase-3 in free DOX-treated cells, the expression of caspase-3 protein in P123@DOX-treated cells was upregulated, and P123-ALN/DP-8@DOX further upregulated caspase-3 expression. The results were consistent with those observed by flow cytometry, suggesting that P123-ALN/DP-8@DOX induced the most apoptosis.

### In vitro bone affinity

**Figure 4:**
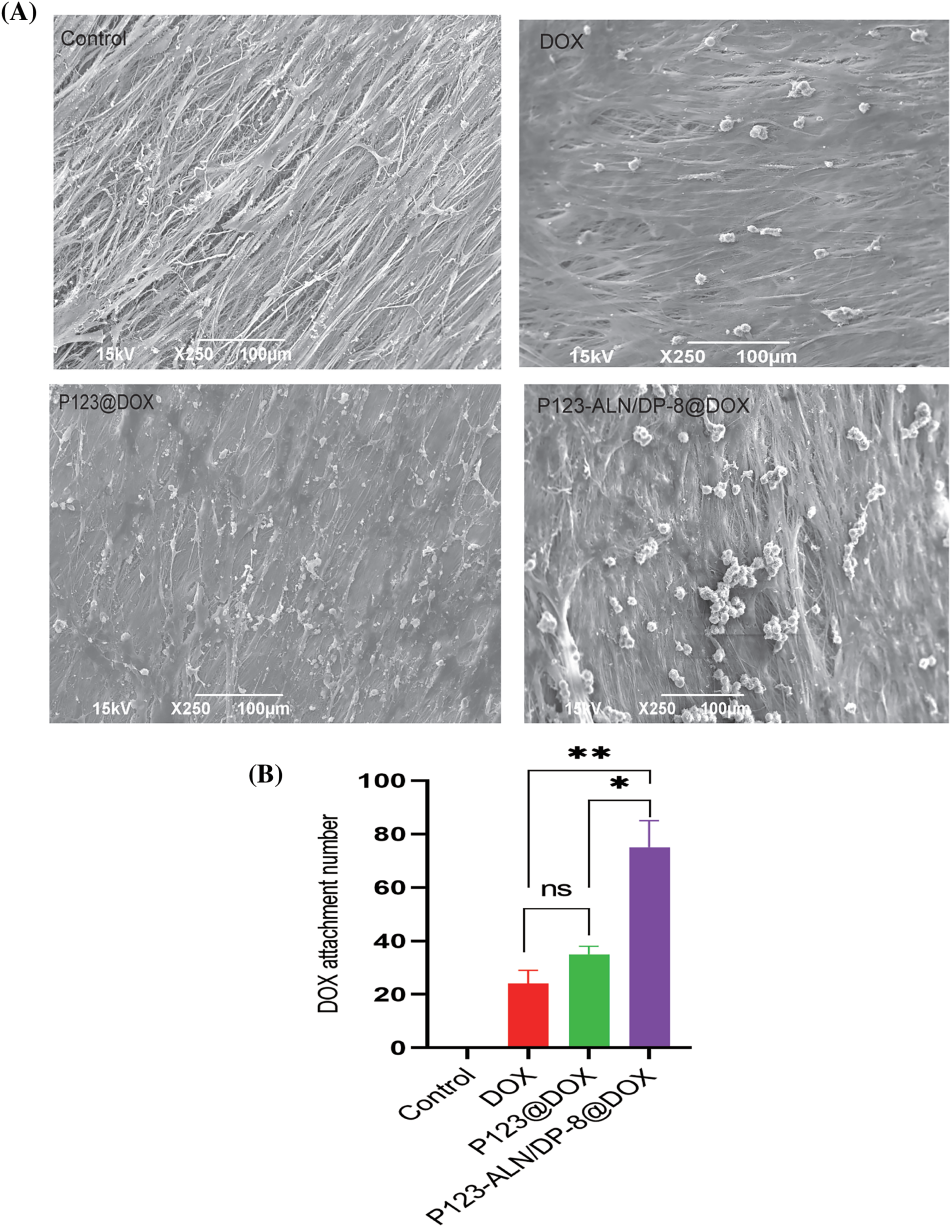
Affinity for bone. Bones were imaged by scanning electron microscopy. Data are expressed as the mean ± SD (n = 3), *p < 0.05, **p < 0.01.

When free DOX, P123@DOX micelles, and P123-ALN/DP-8@DOX micelles were incubated with parietal bones, the adsorption of the micelles was visualized by SEM. Assessment of the surface morphology of bone fibers revealed dense accumulation of P123-ALN/DP-8@DOX micelles on the surface of the bone fibers, while free DOX and P123@DOX micelles were relatively few (Fig. 4). There was no accumulation on the surface of the bone fibers in the control group. These results indicate that ALN played an important role in the adsorption of micelles on the parietal bone surface and that P123-ALN/DP-8@DOX could provide a means to effectively deliver drugs to bone.

### Therapeutic efficacy and bone resorption in an in vitro 3D model of breast cancer bone metastasis

**Figure 5:**
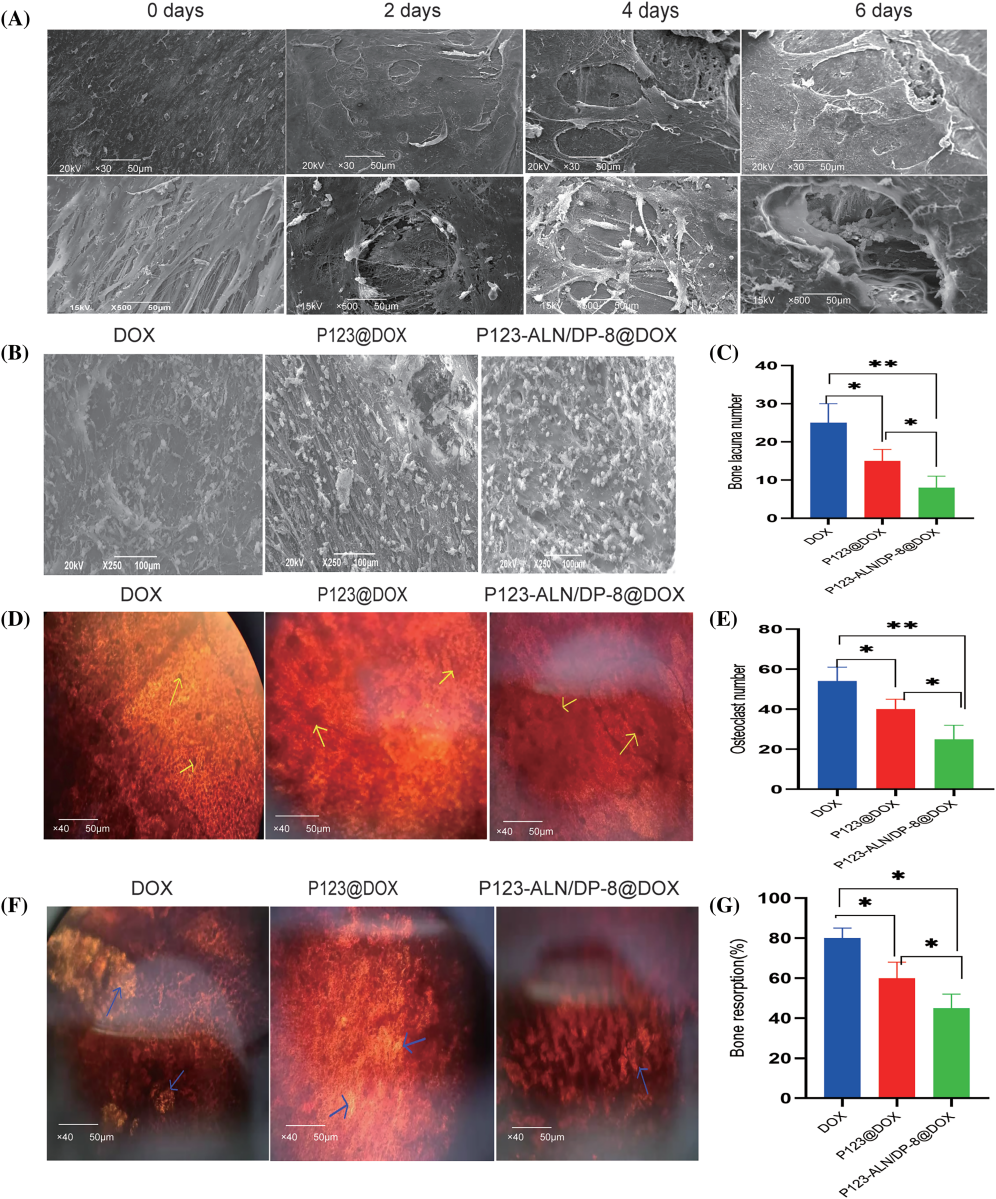
*In vitro* 3D model of breast cancer bone metastasis. (A) Parietal bones from 5-to 7-day-old Sprague Dawley rats were incubated with MDA-MB-231 cells. Tumor cells and bone lacuna appeared on the surface of the bones after different days. The bones were imaged by scanning electron microscopy (SEM). (B) Bone surfaces and (C) Bone lacuna number. (D) Observation of osteoclasts and (E) Osteoclastnumber. (F) Bone resorption areas after incubation with free DOX, P123@DOX, and P123-ALN/DP-8@DOX for 48 h Osteoclasts were stained with neutral red, and are indicated with yellow arrows. The bone resorption areas were counterstained with silver nitrate, and are indicated with green arrows. (G) Bone resorption (%). Data are expressed as the mean ± SD (n = 3), *p < 0.05, **p < 0.01.

The parietal bones of Sprague-Dawley rats aged 5–7 days were incubated with MDA-MB-231 cells to establish an *in vitro* 3D model of breast cancer bone metastasis. Bone resorption and cancer cell adhesion on the parietal surface were observed by SEM. Representative SEM images of the bones are shown in Fig. 5A. The surface of the bones was smooth, and the bone fibers were arranged in an orderly manner in the control. After coincubation with MDA-MB-231 cells for 2, 4, and 6 days, bone lacunas of different depths gradually appeared, and cancer cells attached to the fractured bone fibers around the bone lacunas, indicating that the 3D model of bone metastasis from breast cancer was successfully established.

After the 3D bone metastasis model was incubated with free DOX, P123@DOX, and P123-ALN/DP-8@DOX, the surface of the bones was observed using SEM to evaluate the micelles adhesion, cancer growth and bone resorption. As shown in Figs. 5B and 5C, bones incubated with P123-ALN/DP-8@DOX showed the most accumulation of micelles on the surface of bone fibers and fewer bone lacunae, while the free DOX group and P123@DOX group showed less accumulation on the surface of bone fibers and had larger and deeper bone lacunae. Consistent with the bone-targeting validation of MDA-MB-231 cell coincubation, there was more accumulation of P123-ALN/DP-8@DOX on the bone surfaces in the 3D bone metastasis model. This result indicated that, as a result of ALN, P123-ALN/DP-8 @ DOX attached to bone hydroxyapatite binding sites on the surface of reshaped ossified cancer tissue. These results suggested that P123-ALN/DP-8@DOX could inhibit tumor growth and reduce bone resorption.

In the 3D model of breast cancer bone metastases, the effect of free DOX, P123@DOX and P123-ALN/DP-8@DOX on osteoclast activation is shown in Figs. 5D and 5E. The bones were stained with NR to detect osteoclasts. In the free DOX group, there were multiple multinucleated osteoclasts in the bones. The number of osteoclasts was markedly decreased in the P123-ALN/DP-8@DOX group compared to the free DOX group.

The effects of free DOX, P123@DOX, and P123-ALN/DP-8@DOX on bone resorption are shown in Figs. 5F and 5G. The bones were counterstained with silver nitrate to provide a global view of the bone resorption regions, whereby nonreabsorbed areas are black and reabsorbed areas are white. Compared that in the free DOX group, the amount of bone resorption was markedly reduced in the P123-ALN/DP-8@DOX group. The results were complementary to those observed by SEM, suggesting that P123-ALN/DP-8@DOX had a protective effect on bone tissue.

### In vivo tumor targeting and biodistribution

**Figure 6:**
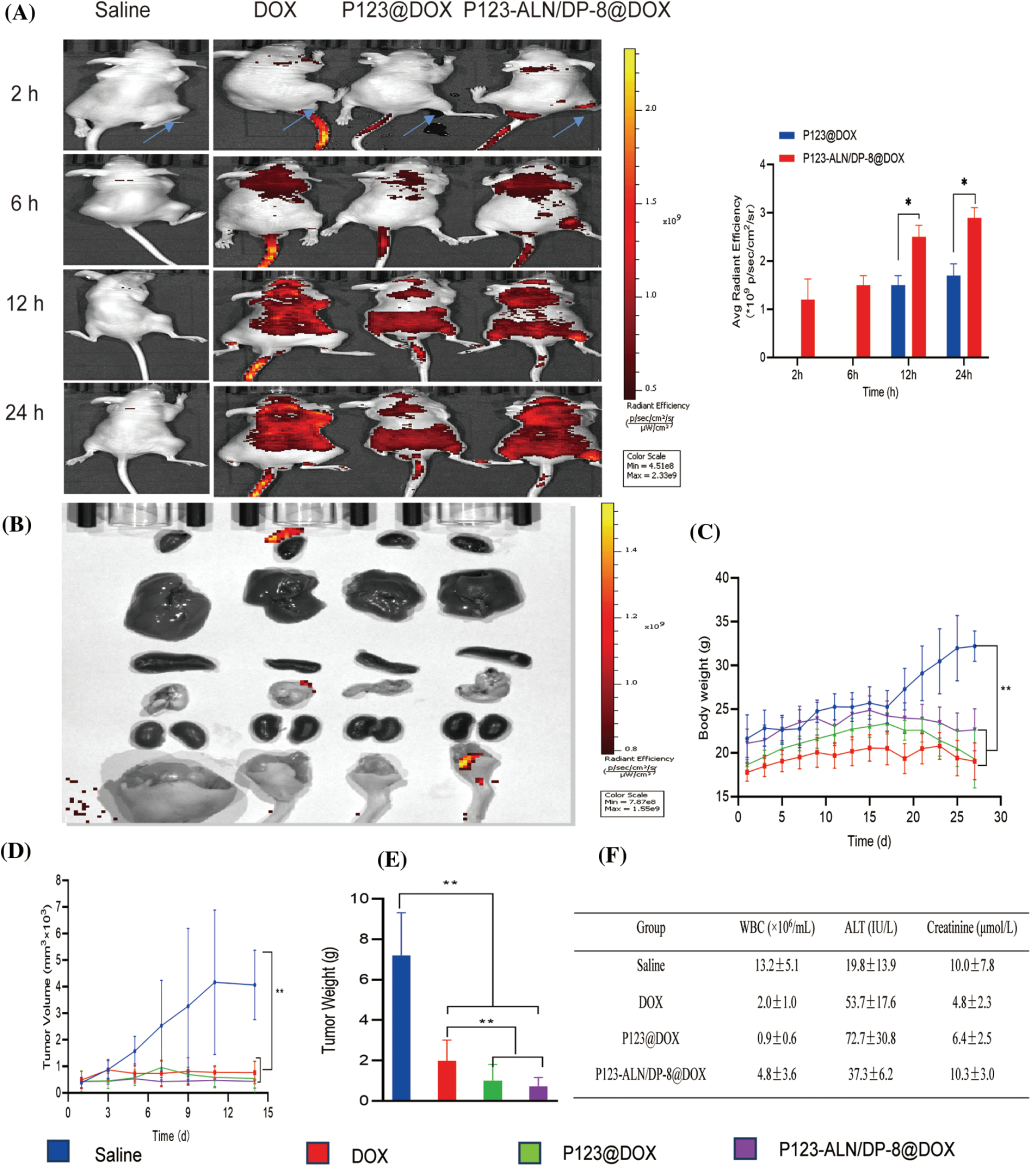
*In vivo* biodistribution and therapeutic efficacy in a murine breast cancer bone metastasis model. Saline (control), free DOX, P123@DOX micelles, and P123-ALN/DP-8@DOX micelles were intravenously administered on days 14 and 21 (DOX dosage of 5 mg/kg). (A) Representative whole body fluorescence images of tumor bearing mice 2, 6, 12, and 24 h after intravenous injection of treatments. (B) Representative fluorescence images of the organs at the end of the experiment. (C) Body weight of mice as a function of time after intratibial MDA-MB-231 cell inoculation and therapy. (D) Tumor volume. (E) Tumor weight at the end of the experiment. (F) Blood chemistry at the end of the experiment. Data are expressed as the mean ± SD (n = 6). *p < 0.05, **p < 0.01.

Research on targeting and *in vivo* biological distribution is essential to evaluate the safety and potential efficacy of drug delivery systems [46]. To further verify the targeting ability of P123-ALN/DP-8@DOX micelles, we used an *in vivo* imaging system to observe DOX distribution *in vivo*. As demonstrated in Fig. 6A, after injection of free DOX into tumor-bearing BALB/c nude mice, red DOX fluorescence was distributed in the body without obvious accumulation in the tumor area. In contrast, after the intravenous injection of P123@DOX micelles at 12 and 24 h, red DOX fluorescence accumulated in the tumor region due to the enhanced permeability and retention effect. The most profound red DOX fluorescence at the tumor region was observed in mice treated with P123-ALN/DP-8@DOX micelles. These results confirm the utility of the conjugation of ALN and DP-8 to P123 micelles to target breast cancer bone metastasis.

### In vivo therapeutic efficacy and safety of P123-ALN/DP-8@DOX micelles

The potential antitumour effect of P123-ALN/DP-8@DOX micelles was evaluated in a bone metastasis model of female BALB/c nude mice inoculated with MDA-MB-231 cells. Fourteen days after inoculation, the mice received the first intravenous injection and 7 days later the second intravenous injection of treatment. After the treatment, tissues and organs were harvested for fluorescence imaging. None of the intervention groups showed DOX accumulation, as shown in Fig. 6B.

Therapeutic efficacy was further evaluated by monitoring body weight and tumor volume, which are known to be negatively affected by the growth of MDA-MB-231 xenografts in mice [47–49]. As illustrated in Figs. 6C–6E, the body weight of the mice in all groups slightly increased during the first 14 days after inoculation. Then, at the start of treatment, the body weight in the control group rapidly increased in the last 14 days, with the tumor volume increasing proportionally. In contrast, the treatment group progressively lost weight over the last 14 days. Compared to mice treated with free DOX and P123@DOX micelles, mice treated with P123-ALN/DP-8@DOX micelles exhibited the least deterioration in body weight and the greatest reduction in tumor volume and tumor weight.

**Figure 7:**
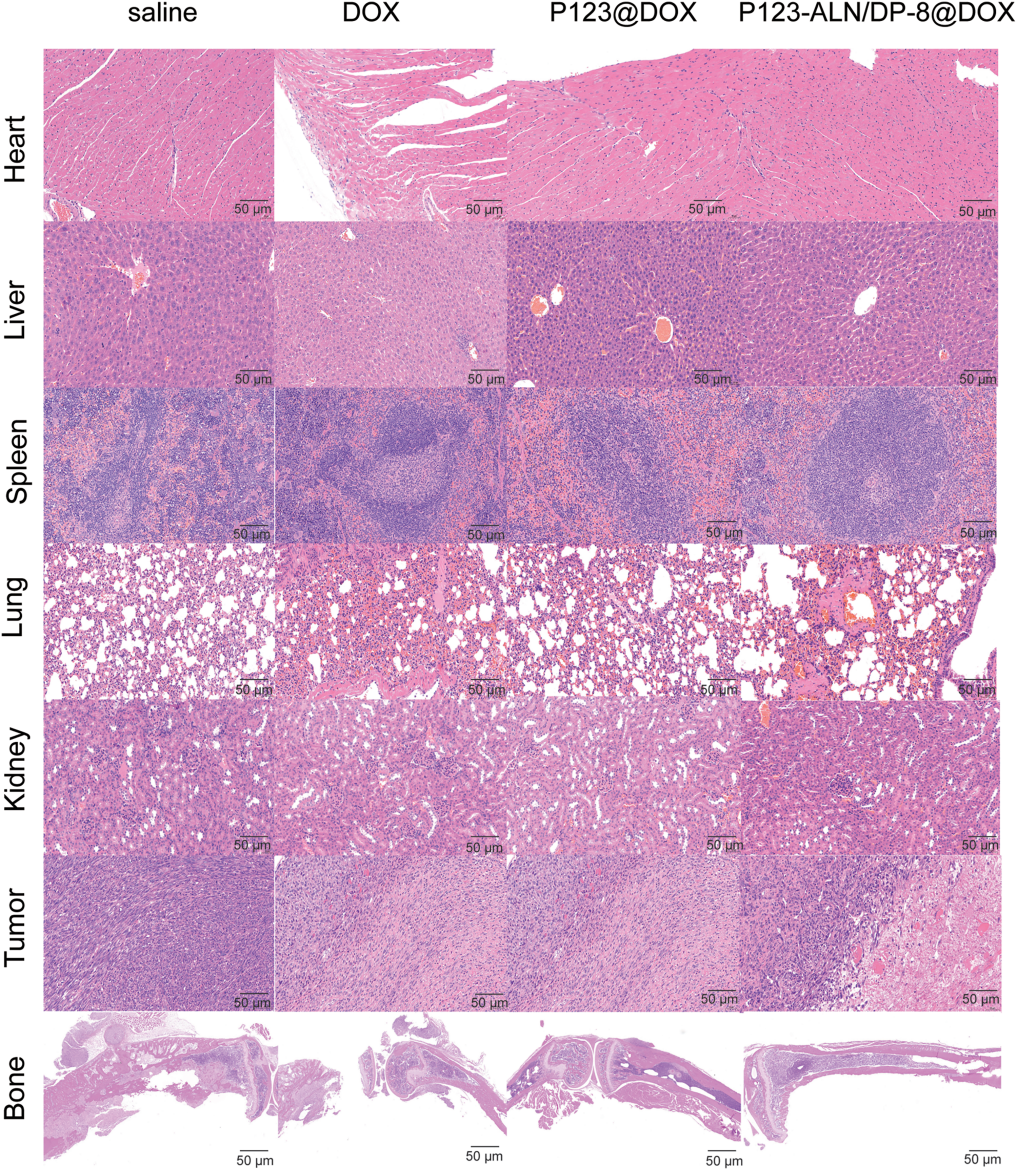
Representative micrographs of histology (H&E staining) in organs and tumor sites (left leg) from breast cancer xenograft model mice following administration of saline, DOX, P123@DOX, and P123-ALN/DP-8@DOX. For all treatments, the DOX concentration was the same (5 mg/kg). None of the collected organs showed acute or chronic inflammation, or apoptotic or necrotic regions. In all groups due to tumor growth the bone showed varying degrees of deformation with the bone in the P123-ALN/DP-8@DOX group being least affected. In the free and micellar DOX groups the bone marrow showed hyperplasia with the bone marrow in the P123-ALN/DP-8@DOX group being least affected. Scale bar = 50 μm.

To detect possible toxicities from the treatments, routine blood analysis and histological studies were performed. The number of WBCs and the levels of ALT and Cr were evaluated to investigate bone marrow suppression and liver and kidney damage, as shown in Fig. 6F. Compared to the control mice, the mice treated with free DOX showed lower WBC numbers and Cr levels and higher ALT levels. Mice treated with P123@DOX showed the most signs of bone marrow suppression and liver and kidney damage. Compared to the P123@DOX group, the P123-ALN/DP-8@DOX group exhibited fairly normal WBC, ALT and Cr values, indicating that P123-ALN/DP-8@DOX caused less damage to bone marrow, liver and kidney functions than free DOX. Histological analysis of the major organs (heart, lungs, liver, kidney, spleen, bone) and tumor tissue sections after completion of the therapeutic regimens was carried out to further assess the therapeutic effect of P123-ALN/DP-8@DOX, and the results are shown in Fig. 7. The morphologies of the major organs were normal, and no areas of acute or chronic inflammation, apoptosis, or necrosis were found in any of the four groups of animals, suggesting that the intervention did not cause adverse effects. Sections of the left tibia showed the largest tumor areas in the control group. The tumor site was smaller and exhibited increased collagen fibers between the cancer cells, more disorderly arranged cancer cells, and cytoplasmic vacuolization in the treatment groups compared to the control group. The smallest tumors were found in the P123-ALN/DP-8@DOX micelle group. The bone in the mice in the control group showed varying degrees of deformation. This deformation was less pronounced in the other groups, especially in the P123-ALN/DP-8@DOX group. Mice treated with free DOX, P123@DOX, and P123-ALN/DP-8@DOX showed bone marrow hyperplasia. The bone marrow of the mice treated with P123-ALN/DP-8@DOX showed the least bone marrow changes compared to the bone marrow of the mice treated with free DOX and P123@DOX groups. The above data implied that P123-ALN/DP-8@DOX induced tumor cell apoptosis and protected the bone tissue from damage.

## Discussion

Bone metastasis is one of the most common complications of malignant tumors. Bone metastasis is a complex and multistep process that is usually formed by a series of dynamic interactions between tumor cells and host cells, causing tumor cells to leave the primary regions and generate distal lesions. Since most patients with bone metastases are in the advanced stages of cancer, the treatment is mainly palliative. In 1986, Pierce first proposed the concept of “bone targeting”, that is, compound molecules that have the ability to selectively bind to bone calcium [50]. This proposal has attracted the attention of many scholars, resulting in new methods for the treatment of bone diseases, such as osteotropic drug delivery systems (ODDSs). Based on this idea, we designed novel bone-targeting micelles (P123-ALN/DP-8@DOX) that used a dual ligand to deliver the traditional antitumor drug doxorubicin to the bone metastasis site to inhibit the resorption of bone and the proliferation of tumor cells. The first ligand, ALN, can target osteoclasts at the metastatic site to inhibit bone resorption. Since the strong adsorption between osteoclasts and ALNs hinders the further delivery of nanoparticles from the bone matrix to tumor cells, the second ligand DP-8 (DMPGTVLP) is added, and the two can collect nanoparticles at the tumor cells through the synergistic effect to further inhibit the proliferation of tumor cells. The second ligand of DMPGTVLP fuses the n-terminal protein (DP-8) of the p8 phage we added, which has proven to have immunogenicity. Importantly, the P123-ALN/DP-8@DOX micelles may pose a potential risk of immunogenicity when used as a therapeutic agent. The ideal bone-targeting agent should have a high bone affinity and be able to release drug properties at the tumor site. We studied the drug release properties of P123-ALN/DP-8@DOX in different pH media. The results showed that the prepared nanoparticles were Ph dependent. Compared with the pH 7.4 releasing medium, a large amount of DOX could be released under the acidic environment of pH 5.0 and 6.5 under the same conditions, indicating that P123-ALN/DP-8@DOX could exist stably in the blood microenvironment. Once it enters the tumor cells, it can be quickly released to kill the tumor cells.

Confocal microscopy and flow cytometry were used to investigate the qualitative and quantitative uptake of P123-ALN/DP-8@DOX by MDA-MB-231 cells. The results showed that P123-ALN/DP-8@DOX had more DOX entering cells than free DOX and P123@DOX, showing a significant time-dependent relationship. Moreover, P123-ALN/DP-8@DOX has a stronger lysosomal escape ability than P123@DOX, which enables more DOX to easily enter the nucleus and enhances the fluorescence intensity in the nucleus, which may be caused by the targeting peptide ligand DP-8. The results were consistent with those of the cytotoxicity test.

Annexin V-FITC/PI double staining was used to investigate the apoptotic effect of P123-ALN/DP-8@DOX on MDA-MB-231 cells. The results showed that the apoptosis rates of P123@DOX and P123-ALN/DP-8@DOX were higher than those of the free DOX group, and the overall apoptosis level was upregulated. The total apoptosis rate of P123-ALN/DP-8@DOX was slightly lower than that of P123@DOX because part of the late apoptosis appeared in the Q1 region. The overall apoptosis rate was decreased, but the results showed that P123-ALN/DP-8@DOX had a significantly stronger effect on promoting apoptosis than free DOX and P123@DOX. At the same time, western blot analysis also proved that P123-ALN/DP-8@DOX could induce an increase in caspase-3 protein content in apoptosis. The mechanism of apoptosis caused by the exogenous caspase 8 pathway or the endogenous caspase 9 pathway needs to be further explored.

MDA-MB-231 cells are the most commonly used cells to study the mechanism of bone metastasis of cancer, and their role in bone tissue is mainly to disrupt the balance between osteoblasts and osteoclasts. Once erodes into bone, factors that promote osteoclast differentiation are produced, such as interleukin-6 (IL6), IL1, prostaglandins, and colony-stimulating factors (CSFs), leading to destruction of the bone matrix. In this study, MDA-MB-231 cells were cocultured with calcaneal bone to simulate the mechanism of cancer-bone interaction under physiological conditions and establish a 3D bone metastasis model. Compared with the control group, the cancer cells colonized the bone surface, and an osteolytic phenomenon occurred. Different depths of bone depression and broken bone fiber were observed everywhere. Free DOX, P123@DOX and P123-ALN/DP-8@DOX were coincubated with calvarial bone, and P123-ALN/DP-8@DOX adhered to the surface of calvarial bone in large quantities. Compared with free DOX and P123@DOX, P123-ALN/-DP-8@DOX had fewer osteoclasts, fewer bone depressions, and a smaller bone resorption area, suggesting that P123-ALN/DP-8@DOX reduced bone resorption by inhibiting the proliferation of MDA-MB-231 cells and reducing the number of osteoclasts.

After the administration of free DOX, P123@DOX and P123-ALN/DP-8@DOX to tumor-bearing nude mice, a large amount of P123-ALN/DP-8@DOX accumulated in the bone tumor site over time, while free DOX was distributed throughout the whole body. During the treatment period, P123-ALN/DP-8@DOX significantly reduced the tumor volume and weight of tumor-bearing nude mice and significantly prolonged the life span of tumor-bearing nude mice.

## Conclusion

P123-ALN/DP-8@DOX has good release properties, has obvious cytotoxicity to MDA-MB-231 cells, enhances the uptake of tumor cells, induces apoptosis, inhibits osteoclast activity, and reduces the bone absorption area. At the same time, tumor growth was significantly inhibited in tumor-bearing nude mice.

## Data Availability

Data available on request from the authors.

## Abbreviations

ALN: Alendronate
BP: Bisphosphonate
BSA: Bovine serum albumin
DOX: Doxorubicin
DMEM: Dulbecco’s modified Eagle’s medium
DP-8: DMPGTVLP fused to the N-terminus of the p8 phage protein
DL: DOX loading
DMSO: Dimethylsulfoxide
DAPI: 4′,6-diamidino-2-phenylindole
EE: Entrapment efficiency
FBS: Fetal bovine serum
FITC: *Fluorescein isothiocyanate*
HA: Hydroxyapatite HE: Hematoxylin-eosin
IC50: 50% inhibition concentration
MTT: 3-(4,5-dinmethyl-2-thiazolyl)-2,5-diphenyl-2Htetr
NHS: N-hydroxysulfosuccinimide sodium salt
NR: Neutral red
OD: Optical density
P123: Pluronic P123
PI: Propidium iodide
RT: Room temperature
SEM: Scanning electron microscopy
SDS: Sodium dodecyl sulfate
TEM: Transmission electron microscopy
